# House Mice in the Atlantic Region: Genetic Signals of Their Human Transport

**DOI:** 10.3390/genes15121645

**Published:** 2024-12-21

**Authors:** Sofia I. Gabriel, Jonathan J. Hughes, Jeremy S. Herman, John F. Baines, Mabel D. Giménez, Melissa M. Gray, Emilie A. Hardouin, Bret A. Payseur, Peter G. Ryan, Alejandro Sánchez-Chardi, Rainer G. Ulrich, Maria da Luz Mathias, Jeremy B. Searle

**Affiliations:** 1 CESAM—Centro de Estudos do Ambiente e do Mar, Departamento de Biologia Animal, Faculdade de Ciências da Universidade de Lisboa, 1749-016 Lisboa, Portugal; mlmathias@ciencias.ulisboa.pt; 2 Department of Evolution, Ecology & Organismal Biology, University of California Riverside, Riverside, CA 92521, USA; jonathan.hughes@ucr.edu; 3 Department of Natural Sciences, National Museums Scotland, Edinburgh EH1 1JF, UK; j.herman@nms.ac.uk; 4 Institute for Experimental Medicine, Kiel University, 24118 Kiel, Germany; j.baines@iem.uni-kiel.de; 5 Max Planck Institute for Evolutionary Biology, 24306 Plön, Germany; 6 IGeHM–Instituto de Genética Humana de Misiones, Parque de la Salud de la Provincia de Misiones “Dr. Ramón Madariaga”, CONICET, Posadas N3300KAZ, Argentina; mdg205@yahoo.com.ar; 7 Facultad de Ciencias Exactas, Químicas y Naturales, Universidad Nacional de Misiones, Posadas N3300LQH, Argentina; 8 Laboratory of Genetics, University of Wisconsin-Madison, Madison, WI 53706, USA; mgray@exactsciences.com (M.M.G.); bret.payseur@wisc.edu (B.A.P.); 9 Department of Life and Environmental Sciences, Bournemouth University, Poole BH12 5BB, UK; ehardouin@bournemouth.ac.uk; 10 FitzPatrick Institute of African Ornithology, University of Cape Town, Rondebosch 7701, South Africa; pryan31@gmail.com; 11 Departament de Biologia Evolutiva, Ecologia i Ciències Ambientals, Universitat de Barcelona, 08028 Barcelona, Spain; alejandro.sanchez.chardi@uab.cat; 12 Institute of Novel and Emerging Infectious Diseases, Friedrich-Loeffler-Institut, Federal Research Institute for Animal Health, 17493 Greifswald-Insel Riems, Germany; rainer.ulrich@fli.de; 13 Partner Site Hamburg-Lübeck-Borstel-Riems, German Centre for Infection Research (DZIF), 17493 Greifswald-Insel Riems, Germany; 14 Department of Ecology and Evolutionary Biology, Cornell University, Ithaca, NY 14853, USA

**Keywords:** Age of Discovery, colonization history, D-loop, *Mus musculus domesticus*, phylogeography, Vikings

## Abstract

Background/Objectives: The colonization history of house mice reflects the maritime history of humans that passively transported them worldwide. We investigated western house mouse colonization in the Atlantic region through studies of mitochondrial D-loop DNA sequences from modern specimens. Methods: We assembled a dataset of 758 haplotypes derived from 2765 mice from 47 countries/oceanic archipelagos (a combination of new and published data). Our maximum likelihood phylogeny recovered five previously identified clades, and we used the haplotype affinities within the phylogeny to infer house mouse colonization history, employing statistical tests and indices. From human history, we predefined four European source areas for mice in the Atlantic region (Northern Europe excluding Scandinavia, Southern Europe, Scandinavia, and Macaronesia) and we investigated the colonization from these source areas to different geographic areas in the Atlantic region. Results: Our inferences suggest mouse colonization of Scandinavia itself from Northern Europe, and Macaronesia from both Southern Europe and Scandinavia/Germany (the latter likely representing the transport of mice by Vikings). Mice on North Atlantic islands apparently derive primarily from Scandinavia, while for South Atlantic islands, North America, and Sub-Saharan Africa, the clearest source is Northern Europe, although mice on South Atlantic islands also had genetic inputs from Macaronesia and Southern Europe (for Tristan da Cunha). Macaronesia was a stopover for Atlantic voyages, creating an opportunity for mouse infestation. Mice in Latin America also apparently had multiple colonization sources, with a strong Southern European signal but also input from Northern Europe and/or Macaronesia. Conclusions: D-loop sequences help discern the broad-scale colonization history of house mice and new perspectives on human history.

## 1. Introduction

‘See the mice in their million hordes from Ibiza to the Norfolk Broads’—David Bowie, Life on Mars, Hunky Dory, 1971

Wherever humans go, they transport (actively or passively) other organisms with them. This includes anthrodependents, i.e., free-living organisms generally dependent on a human environment [1,2,3,4,5]. Alongside written history, material artifacts, and human genetics, archeological and genetic studies on such anthrodependents can inform on the movement history of both the anthrodependents and the humans that transported them [6,7,8,9,10,11,12].

Here, we report on an anthrodependent taxon studied in this way, the western house mouse *Mus musculus domesticus*, whose broad distribution worldwide largely reflects past passive transport by humans [13,14,15,16,17]. Archeology indicates that western house mice were native to the Near East and first associated with humans in the Fertile Crescent about 15,000 years ago [14,18,19,20,21], later expanding into Europe/North Africa, reaching Western Europe via the Mediterranean about 3000 years ago [22,23].

From Western Europe, the western house mouse spread with humans over the whole Atlantic region (our focus here), both to the oceanic islands and continental landmasses neighboring the Atlantic Ocean [15,24,25,26]. This transport around the Atlantic Ocean reflects the movement history of Western European people, with mice as stowaways on maritime vessels. Genetics has already informed us about the Viking and ‘Age of Discovery’ transport of mice to Atlantic islands [27,28,29,30]. For source areas of the colonization of the Atlantic region, here, we focus on genetically typed mice from all European countries on the Atlantic seaboard and Italy. For the colonized areas of the Atlantic region, we incorporate data from all continents surrounding the Atlantic Ocean and the islands within it. As a genetic marker, we use the mitochondrial D-loop sequence, already selected in 1993 for studying the colonization history of house mice, because of its variability and ease of typing [31,32,33], spawning numerous studies thereafter, including ‘ground-truthing’ with ancient DNA [29,34,35]. Particularly for islands, D-loop, as a maternally inherited marker, appears to record first colonization, presumably reflecting a difficulty for incoming female mice to displace residents, thereby enhancing the house mouse as a bioproxy for human history [27,36]. For the Atlantic region, as we delimit it, there are published D-loop data on 2297 western house mice amounting to 693 haplotypes from 33 countries and oceanic island systems (archipelagos and systems with only a single main island). We here add data on 468 individuals and 65 new haplotypes from 27 countries/island systems (14 new), permitting an unusually detailed study of mouse colonization of the Atlantic region. Even though the western house mouse is a well-studied evolutionary system [37,38,39,40,41,42,43], there are no other genetic markers that can match this geographic coverage. Here, we conduct a phylogenetic analysis of all western house mouse haplotypes from the Atlantic region and relate that phylogeny to geography. The data cannot provide the sort of sophisticated analysis possible with genomic data [44,45,46,47], but they generate a compelling broad-brush picture of the human-mediated colonization history of the house mouse in the Atlantic region over the last millennium. In terms of numbers of individuals and geographic spread, this is the largest phylogeographic study carried out on the house mouse and one of the largest such studies of human-mediated transport of an invasive vertebrate.

## 2. Materials and Methods

### 2.1. New Samples

The 468 new western house mouse ‘samples’ obtained were tissue samples, DNA samples, or unpublished D-loop sequences from our laboratory collections or provided by colleagues and museums (see Acknowledgements). For tissue samples (pieces of liver, tail, or feet), genomic DNA was extracted using the DNeasy Blood & Tissue Extraction Kit (Qiagen, Hilden, Germany), following the manufacturer’s guidelines. Dried skin samples obtained from museum collections were soaked in water overnight at 37 °C and extracted with the same kit. New D-loop sequences were obtained by PCR amplification, purification, and sequencing (Text S1). The resulting sequences were shortened to nucleotide positions 15,424–16,276 of the reference mitogenome sequence [48] (i.e., an 840–865 base pair fragment, varying according to indels) to allow alignment with previously published house mouse haplotype sequences. Location data for the new sequences are provided in Table S1.

### 2.2. Sequence Analysis

All DNA sequence traces were checked in Sequencher v. 4.5 (Gene Codes Corp., Ann Arbor, MI, USA) and aligned by eye on Bioedit v7.1.3.0 [49] along with sequences from the literature (Text S2). D-loop haplotypes were obtained with DnaSP ver. 5.10.01 [50], with each distinct haplotype numbered dom_1_–dom_n_.

A rooted maximum likelihood phylogeny [51] based on 758 D-loop haplotypes was generated with IQ-TREE 2 v2.2.0 [52], deploying default search parameters and previously used outgroups [30]. ModelFinder [53] was used to determine the best-fit substitution model (HKY+F+I+R3). For branch support, we applied 1000 replicates of the ultrafast bootstrap approximation [54]. All new and previously published sequences (2765 in total) were assigned to a country or island system, and this information was used for summarizing data for haplotypes and clades that emerged from the phylogenetic analysis. As our study relates to human history, we consider ‘country’ an appropriate geographic identifier. Table S2 lists the countries/island systems included in this study and their classification into areas, given in bold in the justification below.

We classified Europe into geographically coherent source areas reflecting their roles in European maritime exploration and settlement of the Atlantic region [55,56,57]. **Southern Europe** (Italy, Portugal, Spain) was the first area involved in the Age of Discovery exploration, followed later by **Northern Europe** (excluding Scandinavia and here represented by France, Germany, Ireland, the Isle of Man, Luxembourg, Netherlands, and the UK). Even earlier than the Age of Discovery, **Scandinavia** (Denmark, Norway, Sweden) was involved in Viking exploration and settlement of the North Atlantic. Scandinavia was also colonized by house mice from further south, so is both a source area and a colonized area. The same applies to **Macaronesia**, a group of archipelagos (Azores, Cabo Verde, Canary Islands, Madeira Islands) discovered and settled from Western Europe but themselves potentially jumping off points for mice colonizing other parts of the Atlantic region. In terms of areas colonized, the continental areas we identified were **North America** (Canada, USA), **Latin America** (elsewhere in mainland areas of the Americas—Central and South America), and **Sub-Saharan Africa**. Considering human history in broad terms, North America had a Northern European history of colonization different from the Southern European colonization of Latin America. North Africa was not included in this study because its colonization by house mice has already been well described [58], and it was not as important as Western Europe for maritime movements around the Atlantic Ocean. The other colonized areas that we designated were islands in the **North Atlantic** (Faroe, Greenland, Iceland), the **Caribbean** (Guadeloupe, Martinique), and the **South Atlantic** (Falkland [Malvinas] Islands, Gough Island, South Georgia, Tristan da Cunha). We also included sub-Antarctic Marion Island (southwest Indian Ocean) in the latter because it is not far outside the South Atlantic (at the scale that we are working) and is reasonably considered in the same domain in historical terms.

To determine the colonization history of the house mouse, we adopted several approaches. First, because our phylogenetic analysis revealed a number of distinct clades (see Results and Discussion), it allowed us to make a comparison of the prevalence of those clades in the potential source areas (Northern Europe, Southern Europe, Scandinavia, Macaronesia) and all the different colonized areas (Scandinavia, Macaronesia, North Atlantic, North America, Latin America, Caribbean, South Atlantic, Sub-Saharan Africa). The similarity in the clade constitution can help identify which area was the source of house mice in any particular colonized area. We measured the prevalence both in terms of the number of distinct haplotypes and the number of individuals within different clades and used chi-squared tests of association [59] to determine where the data for each potential colonization source best match the data for a particular colonized area. In this way, we examined different aspects of similarity between the colonized area and its potential sources. To satisfy the requirements of the chi-squared test, clades sometimes needed to be merged, reducing degrees of freedom. In some cases, there was a choice as to which clades were combined—the merging that was chosen was always that which generated the highest chi-squared value. Clade merging could mask the differences between the two areas being compared, and so this rule minimized that bias.

Second, we identified all those haplotypes that are found in more than one country/island system, including those found in multiple geographical areas (out of Northern Europe, Southern Europe, Scandinavia, Macaronesia, North Atlantic, North America, Latin America, Caribbean, South Atlantic, Sub-Saharan Africa). These ‘multi-location haplotypes’ can provide valuable supporting evidence that house mice in a particular colonized area derive from a certain source area. Data on individual multi-location haplotypes were inspected for such associations. We also collated all the multi-location haplotypes that are found in each of the four potential source areas (Northern Europe, Southern Europe, Scandinavia, Macaronesia) and determined what proportion of those haplotypes are found in each of the colonized areas (Scandinavia, Macaronesia, North Atlantic, North America, Latin America, Caribbean, South Atlantic, Sub-Saharan Africa). This provides another index for the association of particular source areas with particular colonized areas, a further line of evidence in inferring colonization history.

## 3. Results and Discussion

**Figure 1:**
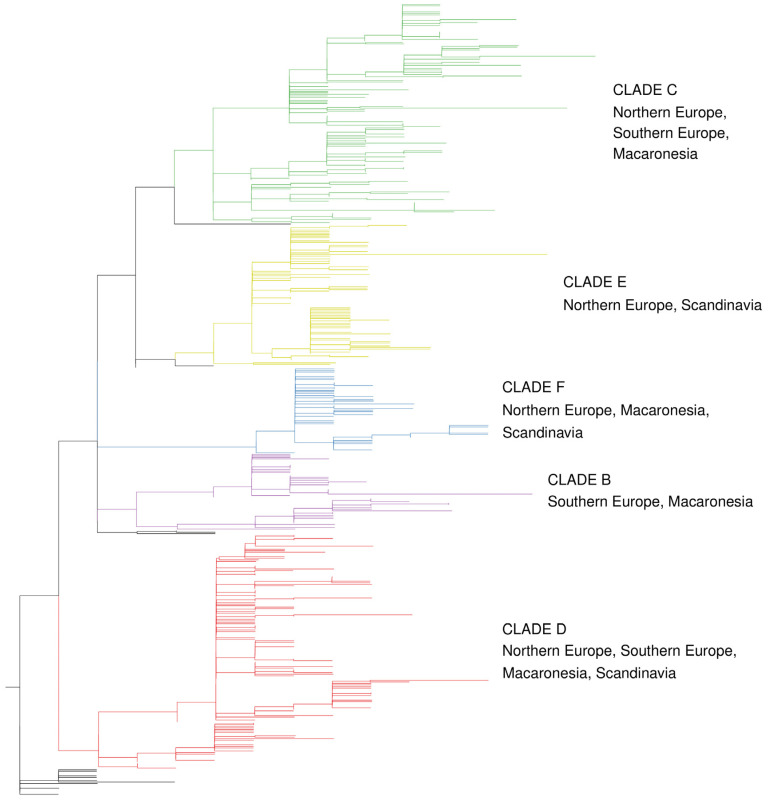
Summary phylogenetic tree for all house mouse haplotypes under consideration, highlighting the particular source areas for Atlantic colonization associated with each of the five previously named clades (B–F) found in the region. The outgroups and the haplotypes that could not be attributed with confidence to a previously named clade had their branches colored black. The naming of the geographic areas follows the convention in this paper. See Figures S1 and S2 for the full tree (including branch support) and further explanation. The phylogeny is based on our new sequences (Table S1) and previously published sequences (Text S2). Particular sequences of importance for interpretation (see Section 3.2 below) are presented in Table S3 (with subsidiary information in Table S2).

As in previous phylogenetic analyses of western house mouse D-loop sequences [e.g., [30]], there is structure to our phylogeny (Figure 1) but low support for individual branches (Figure S1). We have retrieved the same clades as in our previous analyses of D-loop variation [30,60] and retain their designations ‘B’–‘F’. Clade ‘A’, which was found in some previous analyses, and associated with the Near East and Eastern Mediterranean, was not evident in our study.

### 3.1. Clade Occurrence over Broad Geographic Areas

The four predefined source areas differ from each other in clade characteristics (Table 1; Figure 1). Northern Europe and Scandinavia lack clade B, while Southern Europe and Macaronesia are depleted for clade E. Southern Europe also has little representation of clade F, and Scandinavia has little representation of clade C. Both Scandinavia and Macaronesia have a particularly high representation of clade D. Based on *p*-values from chi-squared tests, the source areas differ significantly (Table 2), but the following pairwise combinations are the least divergent: Northern Europe and Scandinavia, Southern Europe and Macaronesia, and Scandinavia and Macaronesia. This is not surprising given the previously published D-loop evidence of mouse colonization of Scandinavia from Northern Europe [31,61,62,63] and Macaronesia from Southern Europe [30] and D-loop and archeological evidence that Vikings from Scandinavia/Germany transported mice to Macaronesia [27,30,64,65].

**Figure 2:**
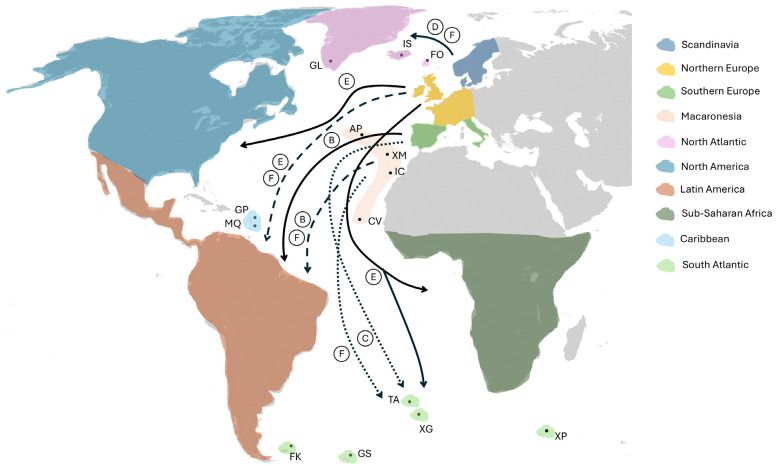
Inferred colonization history of house mice in the Atlantic region from the four defined source areas (Northern Europe, Southern Europe, Scandinavia, Macaronesia) to the five defined colonized areas (North Atlantic, North America, Latin America, South Atlantic, Sub-Saharan Africa). Solid arrows indicate the main routes of colonization. For Latin America, there is a major signal of mouse colonization from Southern Europe but also data suggesting at least partial derivation from Macaronesia and/or Northern Europe (shown with dashed arrows). For the South Atlantic islands, the clade analysis indicates the pre-eminence of Northern Europe in mouse colonization, but multi-location haplotypes support the involvement of Macaronesia and Southern Europe as well (dotted arrows). The most indicative clades present in the source and colonized areas for each of these linkages are shown within circles. There are insufficient data to infer the colonization history of the Caribbean by house mice. The coloring used here does not relate to the coloring in the phylogenetic trees. AP: Azores, CV: Cabo Verde, FK: Falkland (Malvinas) Islands, FO: Faroe, GL: Greenland, GP: Guadeloupe, GS: South Georgia, IC: Canary Islands, IS: Iceland, MQ: Martinique, TA: Tristan da Cunha, XG: Gough Island, XM: Madeira, XP: Marion Island.

Considering wider colonization (Figure 2), Northern Europe shows similar clade characteristics to North America, the South Atlantic, and Sub-Saharan Africa, with clade E well represented and clade B poorly represented (Table 1). Both for the number of haplotypes per clade and the number of individuals per clade, the chi-squared test results show that Northern Europe is less significantly different from North America, the South Atlantic, and Sub-Saharan Africa compared with the other possible source areas (Table 2). The D-loop characteristics of mice from Canada and the USA reflect well the human history of the involvement of the UK and France in the European settlement of North America [66]. Likewise, the South Atlantic islands are British dependencies or are historically associated with the UK, although Tristan da Cunha differs from the other islands in this region by being initially discovered by the Portuguese [67,68]. Sub-Saharan Africa is interesting because initial coastal exploration and some settlements were from Southern Europe (primarily Portugal), but most colonial settlements were from Northern Europe (UK, France, Germany, the Netherlands, Belgium) [69,70]. All these D-loop results match previous mouse genetic studies with various markers [30,36,71,72,73,74].

The North Atlantic islands show the closest clade characteristics with Scandinavia with poor representation of clades B and C, and D is the best represented, followed by F, based on the number of individuals per clade (Table 1). Scandinavia as the source area for the North Atlantic islands (Figure 2) is consistent with the chi-squared tests (Table 2) and is expected from the Viking settlement of Faroe, Iceland, and Greenland and previous mouse D-loop analyses [28,29,63].

As expected, from the human history of Spanish, Portuguese, and Italian pre-eminence in Latin America [75,76], Latin American mice resemble Southern European mice more than Northern European—in particular, clade B is common (Table 1). However, clade F is well represented in Latin American mice but is rare in those from Southern Europe. Clade F is reasonably common in Macaronesian and Scandinavian mice, helping to explain the relatively low chi-squared values in their comparisons with Latin American mice (Table 2) (clade B is also present in Macaronesian mice). Thus, there is the intriguing possibility that the D-loop characteristics of Latin American mice at least partially reflect stowaways picked up in Macaronesia en route between Southern Europe and Latin America (Figure 2). Of the Macaronesian archipelagos, in particular Madeira and the Azores were important as staging posts for the explorers and early settlers of Latin America [77]. These islands may have had high densities of mice due to the absence of competing species and predators, reflecting the ‘island syndrome’ [78,79]. Other scenarios could explain the clade F occurrence in Latin American mice, such as primary Southern European and partial Northern European colonization (Figure 2), which aligns also with the presence of clade E in Latin American mice.

Unfortunately, due to low sampling, only four D-loop haplotypes are available for the Caribbean, so little can be said about mouse colonization there.

### 3.2. Geographic Occurrence of Specific Haplotypes

Individual haplotypes shared between source and colonized areas can provide further insights. A previously reported example is three haplotypes (dom650–652) shared between the Azores and Falkland (Malvinas) Islands [30] within a group of ten sequences, including other Azores and Falkland (Malvinas) Islands sequences and two sequences from Ireland (clade F; Figure S1). This tight association indicates the Azores as a colonizing source for the Falkland (Malvinas) Islands (Figure 2). Using the full data of multi-location haplotypes—those found in more than one country/island system (Table S3)—allows generalization from this result. This complete set of multi-location haplotype data adds more support to Macaronesia’s involvement in the colonization of the South Atlantic, contrasting with the clade analysis that emphasized Northern Europe as the colonization source (compare Table 2 and Table 3). Multi-location haplotype data also further the case that Macaronesia and Northern Europe were involved in the colonization of Latin America and are consistent with the clade analysis with regards to Northern Europe as a source area for North America and Sub-Saharan Africa, and Scandinavia as a source area for the North Atlantic (Table 3).

Further consideration of individual multi-location haplotypes (Table 4 and Table S3) informs these connections. Haplotype dom2 (clade E) is particularly frequent on the Falkland (Malvinas) Islands—but it is also well represented in the UK. Taken together with the above-mentioned data (dom650–652: clade F), this pattern indicates that the mice on the Falkland (Malvinas) Islands may have come from both the UK and the Azores (as a stopover between the UK and the Falklands [Malvinas]), which is consistent with the British colonization of the islands (Table 4). That dom2 might be a mouse marker for British colonial history is indicated by its high frequency on Gough Island (another British dependency in the South Atlantic), the USA, Canada, Cameroon (once a British colony in Sub-Saharan Africa), and also New Zealand and Australia (where dom2 is known as domNZ.4 and AUSTRALIA.01, see [35,80,81,82,83,84,85,86]). Some care is needed in interpretation though because dom2 mice could also have been introduced onto the Falklands (Malvinas) and Gough from France and the USA, respectively. Haplotype dom2 has been found frequently in both countries, and the Falklands (Malvinas) were settled early by the French as well as the British [87] (and Spanish [88]), and there were US sealers visiting Gough [89]. The colonial history of Cameroon also involves Germany [70], and two multi-location haplotypes indicate that linkage (dom162, 180: clade E; Table 4).

Another South Atlantic British dependency is Tristan da Cunha, for which we here report the first D-loop sequences (Table S1). The most frequent Tristan haplotype (dom802) was also recovered from Brazil, and together with other Tristan, Brazilian, and Portuguese haplotypes is in a 10-haplotype group of related sequences (within clade C) lacking UK and Macaronesian haplotypes (most easily seen in Figure S2). Tristan da Cunha was first sighted by Portuguese mariners in 1506, and most likely there was landfall by the Portuguese long before the first colonization by Britain in the early 1800s [67,68]. Pollen evidence indicates an anthropogenic influence beginning in the early 1700s [90]. Given the closely related sequences from Tristan, Portugal, and Brazil (a former Portuguese colony), the mouse mitochondrial DNA signal likely reflects early Portuguese visitation to Tristan rather than the later British colonization, with the mice living feral after arrival. Thus, comparing the Falkland (Malvinas) Islands, Gough Island, and Tristan da Cunha, the mice on British South Atlantic dependencies show signals of British colonization (Falklands [Malvinas] and Gough: dom2 in clade E, with proviso above), Portuguese colonization (Tristan: clade C), and Macaronesian colonization (Falklands [Malvinas]: clade F) (Figure 2, Table 4). This adds to other examples of mouse data providing a novel perspective on human history [27], and it is useful because knowledge of human visitations to the South Atlantic islands is fragmentary (e.g., [68]).

The multi-location haplotypes in clade D (Table 4 and Table S3) are of particular interest. It has already been suggested that the sharing of mouse haplotypes between northern continental Europe and Madeira Island (dom25, 26, 32, 33, 163) reflects Viking visitation of Madeira [27,30] (Table 4). This would have been Danish Vikings coming from Northern Germany/Denmark [91], an area from which mice also colonized Sweden and Norway [31,61,63]—as supported by dom25–27, 32, 42, and 163 (Table 4). Haplotype dom25 is particularly interesting because it is very common in Germany, Denmark, Norway, Sweden, and Madeira Island, indicating that it was taken both north to Fennoscandia and also to Madeira (Table 4). This haplotype is also numerous on the Faroe Islands and likely came there with people from Norway, presumably the Norwegian Vikings (the Norse) [28,92] (Figure 2). A haplotype in clade F, dom6, also shows a signal of transport of mice by the Norse, and it is well represented in the British Isles (UK, Ireland), Norway, and Iceland (Figure 2, Table 4), mirroring the origin of Icelandic people from the British Isles and Norway [29,93,94,95].

## 4. Conclusions

This paper presents a broad-brush analysis of the colonization history of the house mouse in the Atlantic region based on D-loop haplotypes of mice collected using different experimental designs by different workers over 30+ years with simplified categorization based on predefined geographical areas. Despite the limitations to this study, our results both fit with expectations from human history and provide new insights. As expected historically, mice from Northern Europe were the main colonists of Scandinavia, North America, and Sub-Saharan Africa, and Scandinavian mice appear to have been the main colonists of North Atlantic islands. Other results are more intriguing. Macaronesian mice are clearly similar to those from Southern Europe, as expected, but also display a signal of Viking transport from Germany/Scandinavia [27,30]. Macaronesia as a stop-over for Atlantic maritime movements [77] appears to have impacted mouse colonization. Thus, different island systems of the South Atlantic show a signal of colonization from Northern Europe, Southern Europe, Macaronesia, or some combination. Latin America clearly has pre-eminent historical linkages with Southern Europe [75,76], and the mice duly show strong indications of that with the clade analysis—but there are also data implicating involvement of Macaronesia and/or Northern Europe. More sequence data are going to allow us to further explore such findings and fill geographic gaps over the Atlantic region (e.g., in the Caribbean). Moreover, in forthcoming work, we intend to expand the geographical coverage of our analyses of house mouse D-loop sequences to include other parts of the global distribution of the species. The analysis that we have conducted so far, and future analyses, are not only of interest in terms of house mouse colonization history and linkages with human history but they may also have applied significance. In particular, house mice and other anthrodependent rodents may harbor zoonotic pathogens and spread them over large geographic areas (e.g., [96]); knowing the source areas of such invasive pathogen-carrying rodents provides an understanding that may help in controlling zoonoses (e.g., [97]).

## Figures and Tables

**Table 1:** Characterization of house mice according to clades B–F (Figure 1), showing the number of haplotypes (**a**) and the number of individuals (**b**) per clade in potential source areas and areas colonized within the Atlantic region. The naming of the geographic areas follows the convention in this paper.

(**a**)					
	**Number of haplotypes per clade**				
	**B**	**C**	**D**	**E**	**F**
**Source areas**					
Northern Europe	0	76	49	62	36
Southern Europe	31	40	23	1	2
**Source/colonized areas**					
Scandinavia	0	2	29	5	7
Macaronesia	10	18	62	1	9
**Colonized areas**					
North Atlantic	0	0	6	5	4
North America	1	4	1	14	2
Latin America	11	4	4	2	6
Caribbean	0	2	0	1	1
South Atlantic	0	3	7	6	7
Sub-Saharan Africa	0	14	1	14	3
(**b**)					
	**Number of individuals per clade**				
	**B**	**C**	**D**	**E**	**F**
**Source areas**					
Northern Europe	0	274	288	334	185
Southern Europe	84	135	39	1	7
**Source/colonized areas**					
Scandinavia	0	2	135	23	47
Macaronesia	47	135	272	1	56
**Colonized areas**					
North Atlantic	0	0	60	20	54
North America	1	32	5	100	3
Latin America	38	7	7	11	17
Caribbean	0	15	0	5	4
South Atlantic	0	33	22	86	9
Sub-Saharan Africa	0	28	2	57	4

**Table 2:** The results for chi-squared tests for independence comparing among potential source areas and between colonized areas and potential source areas, with the naming of the areas following the convention of this paper. Comparisons are based on (**a**) the number of haplotypes per clade and (**b**) the number of individuals per clade (see Table 1). For chi-squared values and degrees of freedom, see Table S4.

**(a)**				
**Comparisons Among Potential Source Areas (*p*-Values)**				
		**Southern Europe**	**Scandinavia**	**Macaronesia**
Northern Europe		1.75 × 10^−22^	1.17 × 10^−8^	2.64 × 10^−18^
Southern Europe			7.53 × 10^−13^	6.12 × 10^−9^
Scandinavia				0.000816
**Comparisons of Colonized Areas and Potential Source Areas (*p*-Values)**				
	**Northern Europe**	**Southern Europe**	**Scandinavia**	**Macaronesia**
Scandinavia	1.17 × 10^−8^	7.53 × 10^−13^	-	0.000816
Macaronesia	2.64 × 10^−18^	6.12 × 10^−9^	0.000816	-
North Atlantic	0.0288	1.00 × 10^−10^	0.0260	0.106
North America	0.000418	6.59 × 10^−15^	1.96 × 10^−7^	8.91 × 10^−16^
Latin America	5.19 × 10^−21^	6.01 × 10^−5^	6.53 × 10^−7^	1.52 × 10^−6^
South Atlantic	0.112	3.62 × 10^−11^	0.0150	1.12 × 10^−6^
Sub-Saharan Africa	0.0288	8.38 × 10^−12^	9.44 × 10^−9^	6.19 × 10^−14^
**(b)**				
**Comparisons Among Potential Source Areas (*p*-Values)**				
		**Southern Europe**	**Scandinavia**	**Macaronesia**
Northern Europe		3.19 × 10^−109^	2.87 × 10^−33^	6.67 × 10^−71^
Southern Europe			1.40 × 10^−66^	6.86 × 10^−35^
Scandinavia				3.66 × 10^−29^
**Comparisons of Colonized Areas and Potential Source Areas (*p*-Values)**				
	**Northern Europe**	**Southern Europe**	**Scandinavia**	**Macaronesia**
Scandinavia	2.87 × 10^−33^	1.40 × 10^−66^	-	3.66 × 10^−29^
Macaronesia	6.67 × 10^−71^	6.86 × 10^−35^	3.66 × 10^−29^	-
North Atlantic	1.57 × 10^−19^	1.86 × 10^−55^	0.000354	1.83 × 10^−36^
North America	1.72 × 10^−22^	5.02 × 10^−54^	8.91 × 10^−50^	2.34 × 10^−94^
Latin America	1.97 × 10^−115^	1.18 × 10^−19^	8.72 × 10^−31^	3.21 × 10^−37^
Caribbean	0.000209	6.53 × 10^−5^	3.59 × 10^−28^	1.29 × 10^−9^
South Atlantic	7.62 × 10^−10^	3.72 × 10^−46^	8.75 × 10^−36^	3.48 × 10^−73^
Sub-Saharan Africa	6.90 × 10^−12^	1.66 × 10^−43^	6.40 × 10^−40^	1.36 × 10^−78^

**Table 3:** Details of occurrence of mouse D-loop haplotypes that have been found in more than one country/island system (‘multi-location haplotypes’; see also Table S3). For all multi-location haplotypes found in each of the four geographic source areas (Northern Europe, Southern Europe, Scandinavia, Macaronesia), we give the proportion of those haplotypes found in a different location (country/island system) in the same area and the proportion found in each of the other geographic areas (out of Northern Europe, Southern Europe, Scandinavia, Macaronesia, North Atlantic, North America, Latin America, Caribbean, South Atlantic, Sub-Saharan Africa). The naming of the geographic areas follows the convention in this paper. Particular multi-location haplotypes can be found in multiple geographic areas, so the proportions in each column can add up to more than 1.

	**Proportion of all multi-location haplotypes found in at least one country/island system in one of the four geographic source areas that are also found in each of the areas named in the rows of the table**			
	**Northern Europe** **(N = 37)**	**Southern Europe** **(N = 25)**	**Scandinavia** **(N = 18)**	**Macaronesia** **(N = 25)**
Northern Europe	0.49	0.56	0.78	0.52
Southern Europe	0.38	0.28	0.33	0.48
Scandinavia	0.35	0.24	0.22	0.36
Macaronesia	0.35	0.48	0.50	0.20
North Atlantic	0.14	0.08	0.28	0.20
North America	0.19	0.08	0.17	0.04
Latin America	0.19	0.12	0.22	0.28
Caribbean	0.11	0.12	0.11	0.08
South Atlantic	0.11	0.08	0.06	0.24
Sub-Saharan Africa	0.22	0.16	0.17	0.16

**Table 4:** Selected details of ‘multi-location haplotypes’—those that have been found in more than one country/island system (see Table S3 for full listing). The number of sequences per location are listed, and the locations are assigned to a geographic area (out of Northern Europe, Southern Europe, Scandinavia, Macaronesia, North Atlantic, North America, Latin America, Caribbean, South Atlantic, Sub-Saharan Africa, as defined in this paper). The haplotype numbering follows that on the phylogenetic tree (Figure S1), and the two-letter codes for each country/island/archipelago follow Table S2. (**a**) Selected haplotypes that illustrate aspects of colonial history, particularly related to the Western European colonization of the South Atlantic islands and Sub-Saharan Africa (see text). (**b**) Selected haplotypes that illustrate the association of Northern Europe, Scandinavia, Macaronesia, and North Atlantic islands (see text). AP, Azores; AR, Argentina; BO, Bolivia; BR, Brazil; CA, Canada; CM, Cameroon; DE, Germany; DK, Denmark; ES, Spain; FK, Falkland (Malvinas) Islands; FO, Faroe Islands; FR, France; GB, UK; GL, Greenland; GP, Guadeloupe; HN, Honduras; IE, Ireland; IS, Iceland; NL, Netherlands; NO, Norway; PT, Portugal; SE, Sweden; SN, Senegal; TA, Tristan da Cunha; US, USA; XG, Gough Island; XM, Madeira; ZA, South Africa.

**(a)**															
**Haplotype**		**dom2**		**dom650**		**dom651**		**dom652**		**dom162**		**dom180**		**dom802**	
**Clade**		**E**		**F**		**F**		**F**		**E**		**E**		**C**	
Northern Europe		DE(7), FR(11), GB(18), NL(1)								DE(1)		DE(1)			
Southern Europe		PT(1)													
Scandinavia		DK(1), NO(6)													
Macaronesia		AP(1)		AP(13)		AP(21)		AP(8)							
North America		CA(18), US(44)													
Latin America		AR(1), BO(9)												BR(1)	
South Atlantic		FK(29),XG(50)		FK(1)		FK(3)		FK(1)						TA(30)	
Sub-Saharan Africa		CM(33), SN(1), ZA(3)								CM(2)		CM(1)			
**(b)**															
**Haplotype**	**dom25**		**dom26**		**dom27**		**dom32**		**dom33**		**dom42**		**dom163**		**dom6**
**Clade**	**D**		**D**		**D**		**D**		**D**		**D**		**D**		**F**
Northern Europe	DE(57), FR(2), GB(1)		DE(11), FR(2)		DE(8), FR(1)		DE(1)		DE(2)		DE(38), FR(1), GB(2), IE(1)		DE(2), NL(1)		FR(14), GB(12), IE(17)
Southern Europe			ES(2)												ES(6)
Scandinavia	DK(14), NO(24), SE(16)		NO(3), SE(15)		NO(1)		SE(2)				NO(5)		SE(4)		NO(20)
Macaronesia	XM(25)		AP(5), XM(17)				XM(8)		XM(5)				XM(2)		XM(2)
North Atlantic	FO(43), GL(2)		FO(1)										FO(7)		IS(40)
Latin America	BR(3), HN(1)														AR(4)
Caribbean															GP(4)
Sub-Saharan Africa															SN(2)

## Data Availability

All new DNA sequences have been added to GenBank, accession numbers PP751342—PP751400.

## Supplementary Materials

The following supporting information can be downloaded at https://www.mdpi.com/article/10.3390/genes15121645/s1, Figure S1: Maximum likelihood phylogenetic tree of all western house mouse D-loop haplotypes for the Atlantic region newly found in this study plus those from the literature (references [30,60] are cited); Figure S2: Rectangular cladogram, produced by the same analysis as Figure S1, presented to help see some of the haplotype relationships; Table S1: Newly sequenced individuals catalogued according to D-loop haplotype and geographic location, with details of number of individuals for each haplotype and location; Table S2: List of all countries and island systems considered in this study because of their presence in the Atlantic region and the occurrence of new or published D-loop sequences of western house mice that could be attributed to them; Table S3: Details of ‘multi-location haplotypes’—those that have been found in more than one country/island system; Table S4: Chi-squared values and degrees of freedom for the test results given in Table 2; Text S1: PCR and sequencing protocol (references [80,98,99] are cited); Text S2: The literature sources of sequences used in this study (references [27,28,29,30,31,32,36,58,60,63,71,72,80,98,100,101,102,103,104,105,106,107,108,109,110,111] are cited).
