# Supplementary material for: House Mice in the Atlantic Region: Genetic Signals of Their Human Transport
Source: Genes (Basel). 2024 Dec 21;15(12):1645. doi: 10.3390/genes15121645 (PMC11675120; doi:10.3390/genes15121645)
Supplement: Supplementary file 1 [file genes-15-01645-s001.zip › genes-3348928-supplementary.pdf]

SUPPLEMENTARY MATERIALS

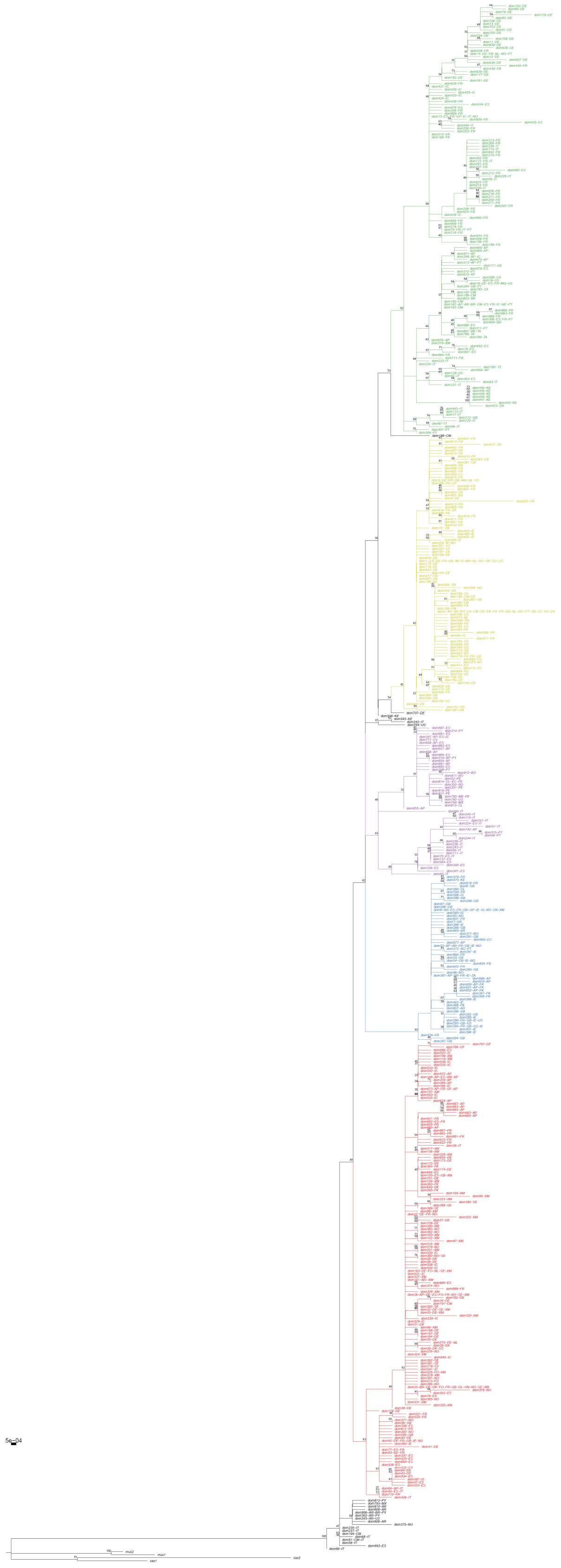

Figure S1. Maximum likelihood phylogenetic tree of all western house mouse D-loop haplotypes for the Atlantic region newly found in this study plus those from the literature (Text S2). Tree generated with IQ-TREE and branch support by ultrafast bootstrap approximation. Geographic distribution of each haplotype indicated by two-letter code for each country or major island/archipelago (Table S2). Figure 1 represents a summary of the tree, without any haplotype names or bootstraps, for easy visualization of the lineages. Figure S2 is another version of the tree, to help visualize some of the haplotype relationships. The clades obtained in this tree could be matched closely to those previously defined in references [30] and [60]. We distinguish those named clades as follows: B: purple, C: green, D: red, E: yellow, F: blue. The other named clade, A, which is associated with the Near East and Eastern Mediterranean, was not evident. Those haplotypes that could not be attributed with confidence to a previously-named clade had their branches colored black. The outgroups (*Mus musculus musculus* and *Mus musculus castaneus*) followed reference [30] and their branches are also colored black.

References as listed in main manuscript:

30. Gabriel, S.I.; Mathias, M.L.; Searle, J.B. Of mice and the 'Age of Discovery': The complex history of colonization of the Azorean archipelago by the house mouse (*Mus musculus*) as revealed by mitochondrial DNA variation. *J. Evol. Biol.* **2015**, *28*, 130–145.
60. Jones, E.P.; Jóhannesdóttir, F.; Gündüz, İ.; Richards, M.B.; Searle, J.B. The expansion of the house mouse into north-western Europe. *J. Zool.* **2011**, *283*, 257–268.

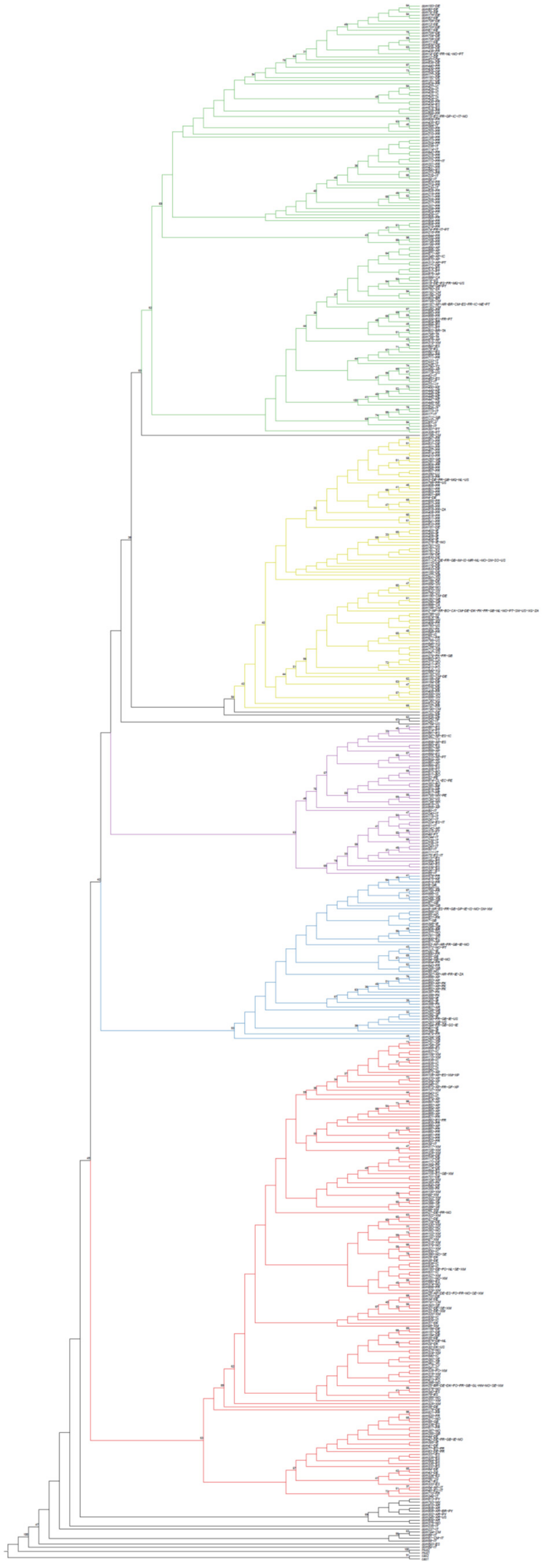

Figure S2. Rectangular cladogram, produced by the same analysis as Figure S1, presented to help see some of the haplotype relationships. Coloring of clades follows Figures 1 and S1.

Table S1. Newly sequenced individuals catalogued according to D-loop haplotype and geographic location, with details of number of individuals for each haplotype and location. The sequences are attributed to a particular country or island system as given in Table S2. Previously unrecorded haplotypes shown in bold. Sequence data submitted to GenBank PP751342—PP751400.

D-loop haplotype	N	Country/Island(s) name	Region	Locality
dom1	4	Germany	North Rhine-Westphalia	Cologne
dom1	4	Germany	Saxony-Anhalt	Magdeburg
dom1	3	Germany	North Rhine-Westphalia	Münster
dom1	4	Germany	North Rhine-Westphalia	Dortmund
dom1	1	Germany	North Rhine-Westphalia	Hamm/Westfalen
dom1	1	France	Grand Est	Moselle
dom1	5	USA	Tennessee	Knoxville
dom2	4	Germany	Saxony-Anhalt	Magdeburg
dom2	11	USA	Arizona	Tucson
dom2	1	USA	Maryland	Southoreast
dom2	2	USA	New Hampshire	Newbury
dom2	6	USA	New Jersey	Princeton
dom2	2	USA	Tennessee	Knoxville
dom2	3	USA	California	Berkeley
dom2	5	USA	California	Windemere, San Ramon
dom2	2	USA	California	Jellys Ferry, Sacramento River
dom2	3	USA	California	Coyote Creek at Rawson Road
dom2	2	USA	California	Oakland
dom2	1	USA	California	Tilden Park
dom2	4	USA	Michigan	Chelsea
*dom2	1	USA	Delaware	Lewes
dom2	1	USA	Maryland	Davidsonville
dom2	2	UK	Oxfordshire	Oxford
dom2	5	UK	Oxfordshire	Abingdon
dom2	1	Cameroon	Southwest Region	Kumba
dom2	5	Bolivia	La Paz Department	La Paz
dom2	1	Senegal	Tambacounda Region	Tambacounda
dom2	2	South Africa	Eastern Cape	
dom2	1	South Africa	Lesotho	
dom3	1	France	Pays de la Loire	Angers
dom3	5	France	Bretagne	Pluvigner
dom3	2	France	Île-de-France	Noisy-le-Grand
dom3	2	France	Provence-Alpes-Côte d'Azur	Camargue
dom3	4	France	Auvergne-Rhône-Alpes	Allier
dom3	5	Martinique		Saint Pierre
dom3	4	USA	Indiana	Indianapolis
dom3	7	USA	Iowa	Spirit Lake

D-loop haplotype	N	Country/Island(s) name	Region	Locality
dom6	2	Spain	Navarra	Agorreta
dom6	4	Spain	Navarra	Eugi
dom6	4	Guadeloupe		Petit Canal
dom6	4	Argentina	Neuquén Province	Zapala
dom6	1	Senegal	Tambacounda Region	Tambacounda
dom6	1	Senegal	Kaolack Region	Kaolack
dom11	1	Germany	North Rhine-Westphalia	Datteln
dom13	27	Germany	Baden-Wuerttemberg	Radolfzell
dom14	19	Germany	Saarland	Saarbrücken
dom15	5	Guadeloupe		Fajou Islet
dom15	4	Guadeloupe		Capesterre
dom15	1	Canary Islands	Tenerife	
dom17	1	Italy	Lombardia	Villa di Tirano
dom18	5	USA	Maryland	Southoreast
dom18	3	USA	Maryland	Hudson Grain Elevator
dom18	2	USA	Maryland	W. Hancock
dom18	1	USA	Maryland	Sun Hill Farm
dom18	2	USA	Iowa	Spirit Lake
dom18	1	Germany	Saarland	Saarbrücken
dom18	1	USA	California	Camp Pendleton
dom18	1	USA	California	East Bank Sacramento River, across from Blue Tent Creek
dom18	2	USA	California	Coyote Creek at Rawson Road
dom18	1	USA	California	Delaware Street, Berkeley
dom18	1	USA	Indiana	Indianapolis
dom18	4	USA	Arizona	Tucson
dom18	6	Martinique		Rocher du Diamant
dom25	1	UK	Oxfordshire	Abingdon
dom25	1	Honduras	Francisco Morazán Department	Comayagüela
dom25	3	Brazil	São Paulo State	Campinas
dom26	1	Spain	Barcelona Region	Les Franqueses
dom30	5	USA	Arizona	Tucson
dom37	2	Germany	Schleswig-Holstein	Helgoland Island
dom53	1	Argentina	Neuquén Province	Zapala
dom53	1	Argentina	Buenos Aires Province	Bahia Blanca
dom64	2	Italy	Calabria	Fiumefreddo
dom128	4	USA	Arizona	Tucson
dom137	1	Spain	Andalusia	Puerto Real, Cádiz
dom187	4	Portugal	Lisbon Region	Mafra
dom187	1	Spain	Catalunya	Torroella de Montgrí
dom187	1	Canary Islands	Gran Canaria	La Garita
dom187	1	Cameroon	Southwest Region	Bota, near Victoria
dom187	1	Brazil	Rio de Janeiro State	Rio de Janeiro

D-loop haplotype	N	Country/Island(s) name	Region	Locality
dom187	2	Argentina	Chubut Province	Puerto Madryn
dom187	1	Argentina	Chubut Province	Esquel
dom290	1	USA	California	Snelling
dom290	1	USA	Arizona	Tucson
dom293	1	USA	Maryland	Ridgely
dom302	2	Argentina	Rio Negro Province	Bariloche
dom302	4	Argentina	Mendoza Province	Luján de Cuyo
dom302	2	Argentina	Buenos Aires Province	Autonomous City of Buenos Aires
dom302	1	Argentina	Corrientes Province	Corrientes
dom302	1	Argentina	Formosa Province	Formosa
dom302	7	Paraguay	Asunción Capital District	Asunción
dom309	1	Spain	Navarra	Agorreta
dom345	5	Argentina	Rio Negro Province	Bariloche
dom345	1	Argentina	Buenos Aires Province	Autonomous City of Buenos Aires
dom345	2	Argentina	Mendoza Province	Mendoza
dom345	2	Argentina	Córdoba Province	Córdoba
dom345	3	USA	Arizona	Tucson
dom346	1	Canary Islands	Gran Canaria	La Garita
dom348	1	Canary Islands	Gran Canaria	La Garita
**dom349**	**1**	**Italy**	**Calabria**	**Fiumefreddo**
**dom350**	**1**	**Luxembourg**		
dom394	1	UK	Cumbria	Lake District
dom397	1	Argentina	Buenos Aires Province	Autonomous City of Buenos Aires
dom397	1	Argentina	Buenos Aires Province	Bahia Blanca
dom397	1	South Africa	Pietermaritzburg, Natal	Midmar Dam Nature Reserve
dom410	1	France	Île-de-France	Paris
dom584	1	Spain	Andalusia	Puerto Real, Cádiz
dom616	1	South Africa	Eastern Cape	
dom640	4	Germany	North Rhine-Westphalia	Cologne
dom673	1	French Guiana	Sinnamary Commune	Sinnamary
**dom690**	**1**	**Spain**	**Catalunya**	**Torroella de Montgrí**
**dom691**	**1**	**Spain**	**Catalunya**	**Torroella de Montgrí**
**dom692**	**1**	**Spain**	**Catalunya**	**Torroella de Montgrí**
**dom693**	**1**	**Spain**	**Barcelona Region**	**Les Franqueses**
**dom694**	**1**	**Spain**	**Barcelona Region**	**Les Franqueses**
**dom695**	**2**	**France**	**Provence-Alpes-Côte d'Azur**	**Hyères**
**dom696**	**5**	**France**	**Bretagne**	**Béniguet Island**
**dom697**	**1**	**France**	**Bretagne**	**Rennes**
**dom698**	**1**	**France**	**Pays de la Loire**	**Angers**
**dom699**	**1**	**France**	**Massif Central**	
**dom700**	**1**	**France**	**Massif Central**	

D-loop haplotype	N	Country/Island(s) name	Region	Locality
**dom701**	**13**	**Germany**	**North Rhine-Westphalia**	**Cologne**
**dom702**	**4**	**Germany**	**Saxony-Anhalt**	**Magdeburg**
**dom703**	**1**	**Germany**	**Baden-Wuerttemberg**	**Radolfzell**
**dom704**	**1**	**Germany**	**Baden-Wuerttemberg**	**Radolfzell**
**dom705**	**4**	**Germany**	**Saarland**	**Saarbrücken**
**dom706**	**1**	**Germany**	**Saarland**	**Saarbrücken**
**dom707**	**1**	**Germany**	**Saarland**	**Saarbrücken**
**dom708**	**1**	**Germany**	**North Rhine-Westphalia**	**Datteln**
**dom709**	**2**	**Germany**	**Schleswig-Holstein**	**Helgoland Island**
**dom710**	**1**	**France**	**Île-de-France**	**Paris**
**dom711**	**2**	**France**	**Provence-Alpes-Côte d'Azur**	**Camargue/Étang de Vaccarès**
**dom712**	**1**	**UK**	**Oxfordshire**	**Oxford**
**dom713**	**1**	**UK**	**Kent**	**Canterbury**
**dom753**	**1**	**USA**	**California**	**Lafayette**
dom764	1	USA	Arizona	Tucson
**dom777**	**1**	**Cabo Verde**	**Santo Antão Island**	
**dom778**	**1**	**Cabo Verde**	**São Nicolau Island**	
**dom780**	**1**	**Tanzania**	**Pemba Island**	**Mkoani**
**dom781**	**1**	**South Africa**	**Western Cape Province**	**De Hoop**
**dom782**	**2**	**South Africa**	**Free State**	
**dom783**	**1**	**USA**	**Arizona**	**Tucson**
**dom784**	**2**	**USA**	**Arizona**	**Tucson**
**dom785**	**2**	**USA**	**Arizona**	**Tucson**
**dom786**	**2**	**USA**	**New Jersey**	**Princeton**
**dom787**	**1**	**USA**	**Tennessee**	**Knoxville**
dom788	1	USA	Indiana	Indianapolis
**dom789**	**2**	**USA**	**Maryland**	**Upper Marlboro**
dom790	1	USA	Maryland	Centreville
**dom791**	**1**	**USA**	**California**	**Bouquet Canyon**
**dom792**	**1**	**USA**	**California**	**Hollenbeck Canyon**
**dom793**	**1**	**Mexico**	**Mexico City Capital District**	**Mexico City**
**dom794**	**2**	**Mexico**	**Baja California**	**Maneadero**
**dom795**	**1**	**Mexico**	**Baja California**	**Maneadero**
**dom795**	**1**	**Peru**	**Huancavelica Province**	**Huancavelica**
**dom796**	**1**	**French Guiana**	**Sinnamary Commune**	**Sinnamary**
**dom797**	**1**	**French Guiana**	**Cayenne Commune**	**Cayenne**
**dom798**	**1**	**Tristan da Cunha**		
**dom799**	**2**	**Tristan da Cunha**		
**dom800**	**1**	**Ecuador**	**Esmeraldas Province**	**Timbre/Chinca**
**dom801**	**1**	**Brazil**	**Marajó Island**	**Caldeirão**
**dom802**	**1**	**Brazil**	**Paraíba**	**Reserva Biológica Guaribas**
**dom802**	**30**	**Tristan da Cunha**		
**dom803**	**1**	**Brazil**	**Pernambuco State**	**Parque Nacional do Catimbau**
**dom804**	**1**	**Brazil**	**Pernambuco State**	**Reserva Biológica de Saltinho**

D-loop haplotype	N	Country/Island(s) name	Region	Locality
**dom805**	**8**	**Brazil**	**Rio de Janeiro State**	**Rio de Janeiro**
**dom806**	**1**	**Brazil**	**Rio Grande do Sul State**	**Erechim**
**dom806**	**1**	**Brazil**	**Rio Grande do Sul State**	**Mato Castelhano**
**dom806**	**1**	**Paraguay**	**Asunción Capital District**	**Asunción**
**dom806**	**6**	**Argentina**	**Buenos Aires Province**	**Bahia Blanca**
**dom806**	**1**	**Argentina**	**Buenos Aires Province**	**Autonomous City of Buenos Aires**
**dom807**	**2**	**Argentina**	**Neuquén Province**	**Zapala**
**dom808**	**2**	**Argentina**	**Buenos Aires Province**	**Autonomous City of Buenos Aires**
**dom808**	**2**	**Argentina**	**La Pampa Province**	**Estancia La Florida, Toay Department**
**dom808**	**1**	**Argentina**	**Buenos Aires Province**	**Bahia Blanca**
**dom809**	**1**	**Argentina**	**Corrientes Province**	**Corrientes**
**dom810**	**1**	**Argentina**	**Misiones Province**	**Azara**
dom811	8	Bolivia	La Paz Department	La Paz
**dom812**	**1**	**Bolivia**	**La Paz Department**	**La Paz**
**dom813**	**1**	**Paraguay**	**Asunción Capital District**	**Asunción**
dom814	9	Chile	Santiago Province	La Pintana
dom814	1	Ecuador	Azuay Province	Laguna Llaviuco
**dom815**	**1**	**Chile**	**Santiago Province**	**La Pintana**

Table S2. List of all countries and island systems considered in this study because of their presence in the Atlantic region and the occurrence of new or published D-loop sequences of western house mice that could be attributed to them. Each is assigned a 2-letter code, either previously recognized, or newly formulated. Each named entity is attributed to a geographic area as defined in this paper (Northern Europe, Southern Europe, Scandinavia, Macaronesia, North Atlantic, North America, Latin America, Caribbean, South Atlantic, Sub-Saharan Africa).

Country/Island(s) name	Ruling country (if relevant)	2-letter code	Area
Azores	Portugal (autonomous region) (UPS CODE)	AP	Macaronesia
Argentina		AR	Latin America
Bolivia		BO	Latin America
Brazil		BR	Latin America
Canada		CA	North America
Chile		CL	Latin America
Cameroon		CM	Sub-Saharan Africa
Cabo Verde		CV	Macaronesia
Germany		DE	Northern Europe
Denmark		DK	Scandinavia
Ecuador		EC	Latin America
Spain		ES	Southern Europe
Falkland Islands (Malvinas Islands)	UK (devolved parliamentary dependency)	FK	South Atlantic
Faroe Islands		FO	North Atlantic
France		FR	Northern Europe
UK		GB	Northern Europe
French Guiana		GF	Latin America
Greenland	Denmark (autonomous territory)	GL	North Atlantic
Guadeloupe	France (overseas department)	GP	Caribbean
South Georgia	UK (overseas territory)	GS	South Atlantic
Honduras		HN	Latin America
Canary Islands	Spain (autonomous community)	IC	Macaronesia
Ireland		IE	Northern Europe
Isle of Man	UK (crown dependency)	IM	Northern Europe
Iceland		IS	North Atlantic
Italy		IT	Southern Europe
Kenya		KE	Sub-Saharan Africa
Luxembourg		LU	Northern Europe
Martinique	France (territorial collectivity)	MQ	Caribbean
Mauritania		MR	Sub-Saharan Africa
Mexico		MX	Latin America
Niger		NE	Sub-Saharan Africa
Netherlands		NL	Northern Europe
Norway		NO	Scandinavia

Country/Island(s) name	Ruling country (if relevant)	2-letter code	Area
Peru		PE	Latin America
Portugal		PT	Southern Europe
Paraguay		PY	Latin America
Sweden		SE	Scandinavia
Senegal		SN	Sub-Saharan Africa
Somalia		SO	Sub-Saharan Africa
Tristan da Cunha	UK (overseas territory)	TA	South Atlantic
Tanzania		TZ	Sub-Saharan Africa
USA		US	North America
Gough Island	UK (overseas territory) (OUR CODE)	XG	South Atlantic
Madeira	Portugal (autonomous region) (OUR CODE)	XM	Macaronesia
Marion Island	South Africa (municipality) (OUR CODE)	XP	South Atlantic
South Africa		ZA	Sub-Saharan Africa

Table S3. Details of 'multi-location haplotypes' – those that have been found in more than one country/island system. Numbers of sequences per location are listed and the locations are assigned to a geographic area (out of Northern Europe, Southern Europe, Scandinavia, Macaronesia, North Atlantic, North America, Latin America, Caribbean, South Atlantic, Sub-Saharan Africa, as defined in this paper). The haplotype numbering follows that on the phylogenetic tree (Figure S1) and the two-letter codes for each country/island/archipelago follow Table S2.

Haplotype	N Europe	S Europe	Scandinavia	Macaronesia	N Atlantic	N America	L America	Caribbean	S Atlantic	S-S Africa
**CLADE B**										
dom75		ES(1), IT(2)								
dom224		ES(4), IT(1)								
dom310		PT(4)		AP(1)						
dom347		ES(7)		AP(15), IC(1)						
dom658		ES(8)		AP(4)						
dom795							MX(1), PE(1)			
dom814							CL(9), EC(1), PE(5)			
**CLADE C**										
dom14	DE(33), FR(1), NL(4)	PT(3)	NO(1)							
dom15	FR(24)	ES(2), IT(1)	NO(1)	IC(1)				GP(9)		
dom18	FR(3), DE(1)	ES(1)				US(24)		MQ(6)		
dom74	FR(1)	IT(3), PT(3)								
dom112	FR(12)	IT(5)								
dom187	FR(8)	ES(12), PT(25)		AP(58), IC(1)			AR(3), BR(1)			CM(11), NE(1)
dom284	GB(1)	PT(2)								
dom309	FR(21)	ES(1), PT(6)								
dom313		PT(2)		AP(2)						
dom348				AP(12), IC(2)						
dom802							BR(1)		TA(30)	
**CLADE D**										
dom25	DE(57), FR(2), GB(1)		DK(14), NO(24), SE(16)	XM(25)	FO(43), GL(2)		BR(3), HN(1)			
dom26	DE(11), FR(2)	ES(2)	NO(3), SE(15)	AP(5), XM(17)	FO(1)					
dom27	DE(8), FR(1)		NO(1)							
dom30			DK(1)			US(5)				
dom32	DE(1)		SE(2)	XM(8)						

Haplotype	N Europe	S Europe	Scandinavia	Macaronesia	N Atlantic	N America	L America	Caribbean	S Atlantic	S-S Africa
dom33	DE(2)			XM(5)						
dom42	DE(38), FR(1), GB(2), IE(1)		NO(5)							
dom45		ES(1), IT(1)								
dom64		IT(3)		AP(5)						
dom77	FR(2)	ES(1)								
dom83	DE(1), FR(2)									
dom101			NO(2)	XM(4)						
dom105	GB(1)	ES(1)		XM(2)						
dom108		ES(3)		AP(32), XM(6)					XP(13)	
dom163	DE(2), NL(1)		SE(4)	XM(2)	FO(7)					
dom326				XM(1)	FO(2)					
dom380			NO(4), SE(1)							
dom575	DE(1), NL(1)									
dom673	FR(1)			AP(1)			GF(1)		XP(1)	
dom682	FR(25)	ES(4)								
**CLADE E**										
dom1	DE(29), FR(7), GB(14), IM(1), NL(6)		NO(3)		IS(2)	CA(1), US(5)				SN(1), SO(1)
dom2	DE(7), FR(11), GB(18), NL(1)	PT(1)	DK(1), NO(6)	AP(1)		CA(18), US(44)	AR(1), BO(9)		FK(29), XG(50)	CM(33), SN(1), ZA(3)
dom3	FR(45), DE(6), GB(1), NL(2)					US(11)		MQ(5)		
dom162	DE(1)									CM(2)
dom180	DE(1)									CM(1)
dom278	IE(8)		NO(10)							
dom279	FR(1), GB(1)								FK(2)	
dom616	FR(52)									ZA(1)
dom788	FR(8)					US(1)				
**CLADE F**										
dom6	FR(14), GB(12), IE(17)	ES(6)	NO(20)	XM(2)	IS(40)		AR(4)	GP(4)		SN(2)
dom53	FR(21), GB(2), IE(1)		NO(6)	AP(3)			AR(2)			

Haplotype	N Europe	S Europe	Scandinavia	Macaronesia	N Atlantic	N America	L America	Caribbean	S Atlantic	S-S Africa
dom54	GB(3), IE(10)		NO(10)							
dom290	FR(1), GB(2), IE(15)					US(2)				
dom293	GB(6)					US(1)				
dom372		PT(1)	NO(1)							
dom394	FR(3), GB(1), IE(3)								GS(1)	
dom397	FR(3), IE(1)			AP(1)			AR(2)			ZA(1)
dom650				AP(13)					FK(1)	
dom651				AP(21)					FK(3)	
dom652				AP(8)					FK(1)	
**NO NAMED CLADE**										
dom61		IT(7)								CM(2)
dom302							AR(12), PY(7)			
dom345						US(3)	AR(11)			
dom806							AR(7), BR(2), PY(1)			

Table S4. Chi-squared values and degrees of freedom for test results given in Table 2. Comparisons among potential source areas and between colonized areas and potential source areas, with naming of the areas following the convention of this paper. Comparisons based on (a) numbers of haplotypes per clade and (b) numbers of individuals per clade (see Table 1).

(**a**)

Comparisons among potential source areas

	**Northern Europe**	**Southern Europe**	**Scandinavia**
Southern Europe	108.2 (4)	-	-
Scandinavia	39.8 (3)	59.5 (3)	-
Macaronesia	88.6 (4)	41.1 (3)	14.2 (2)

Comparisons of colonized areas and potential source areas

	**Northern Europe**	**Southern Europe**	**Scandinavia**	**Macaronesia**
Scandinavia	39.8 (3)	59.5 (3)	-	14.2 (2)
Macaronesia	88.6 (4)	41.1 (3)	14.2 (2)	-
North Atlantic	4.8 (1)	46.0 (2)	5.0 (1)	2.6 (1)
North America	15.6 (2)	65.3 (2)	30.9 (2)	69.3 (2)
Latin America	97.6 (3)	22.2 (3)	28.5 (2)	26.8 (2)
South Atlantic	6.0 (3)	51.6 (3)	8.4 (2)	27.4 (2)
Sub-Saharan Africa	9.0 (3)	54.6 (3)	40.2 (3)	64.6 (3)

(**b**)

Comparisons among potential source areas

	**Northern Europe**	**Southern Europe**	**Scandinavia**
Southern Europe	510.7 (4)	-	-
Scandinavia	154.5 (3)	313.4 (4)	-
Macaronesia	333.4 (4)	162.0 (3)	139.5 (4)

Comparisons of colonized areas and potential source areas

	**Northern Europe**	**Southern Europe**	**Scandinavia**	**Macaronesia**
Scandinavia	154.5 (3)	313.4 (4)	-	139.5 (4)
Macaronesia	333.4 (4)	162.0 (3)	139.5 (4)	-
North Atlantic	90.7 (3)	261.8 (4)	15.9 (2)	173.5 (4)
North America	104.4 (3)	255.2 (4)	230.9 (3)	442.0 (4)
Latin America	539.4 (4)	94.9 (4)	147.1 (4)	177.1 (4)
Caribbean	19.6 (3)	19.3 (2)	126.4 (2)	40.9 (2)
South Atlantic	45.4 (3)	218.6 (4)	166.1 (3)	344.0 (4)
Sub-Saharan Africa	55.0 (3)	206.3 (4)	185.3 (3)	369.0 (4)

Text S1. PCR and sequencing protocol

We used the following protocol to obtain new sequences from extracted DNA. The mitochondrial DNA D-loop and flanking tRNAs were amplified by PCR using the primer pair L15774 (5′TGA ATT GGA GGA CAA CCA GT 3′) and H2228 (5′ TTA TAA GGC CAG GAC CAA AC 3′) as described in reference [98].

Rigorous contamination controls were used for museum-curated specimens [99]. The DNA recovered from most museum samples was degraded requiring amplification with three published primer pairs [80] producing overlapping fragments: dloopF1 – 5′GCA CCC AAA GCT GGT ATT CT 3′/ dloopR1 – 5′TTG TTG GTT TCA CGG AGG AT 3′; dloopF2 – 5′ACT ATC CCC TTC CCC ATT TG 3′/ dloopR2 – 5′ GAT TGG GTT TTG CGG ACT AA 3′; dloopF3 – 5′ATA GCC GTC AAG GCA TGA AA 3′/ dloopR3 – 5′GCT TTG CTT TGT TAT TAA GCT ACA 3′. Samples that failed to amplify any of these fragments were discarded.

Successful PCR products were purified with the QIAquick PCR Purification Kit (Qiagen, Hilden, Germany) and sequenced commercially in both directions using the PCR primers (Macrogen Inc., Seoul, South Korea).

References as listed in main manuscript:

80. Gabriel, S.I.; Stevens, M.I.; Mathias, M.D.; Searle, J.B. Of mice and 'convicts': origin of the Australian house mouse, *Mus musculus*. *PLoS One* **2011**, *6*, e28622.
98. Searle, J.B.; Jones, C.S.; Gündüz, İ.; Scascitelli, M.; Jones, E.P.; Herman, J.S.; Rambau, R.V.; Noble, L.R.; Berry, R.J.; Giménez, M.D.; Jóhannesdóttir, F. Of mice and (Viking?) men: phylogeography of British and Irish house mice. *Proc. R. Soc. B* **2009**, *276*, 201–207.
99. Martínková, N.; Searle, J.B. Amplification success rate of DNA from museum skin collections: a case study of stoats from 18 museums. *Mol. Ecol. Notes* **2006**, *6*, 1014–1017.

Text S2. The literature sources of sequences used in this study

References as listed in main manuscript:

27. Förster, D.W.; Gündüz, İ.; Nunes, A.C.; Gabriel, S.; Ramalhinho, M.G.; Mathias, M.L.; Britton-Davidian, J.; Searle, J.B. Molecular insights into the colonization and chromosomal diversification of Madeiran house mice. *Mol. Ecol.* **2009**, *18*, 4477–4494.
28. Jones, E.P.; Jensen, J.-K.; Magnussen, E.; Gregersen, N.; Hansen, H.S.; Searle, J.B. A molecular characterization of the charismatic Faroe house mouse. *Biol. J. Linn. Soc.* **2011**, *102*, 471–482.
29. Jones, E.P.; Skirnisson, K.; McGovern, T.H.; Gilbert, M.T.P.; Willerslev, E.; Searle, J.B. Fellow travellers: A concordance of colonization patterns between mice and men in the North Atlantic region. *BMC Evol. Biol.* **2012**, *12*, 35.
30. Gabriel, S.I.; Mathias, M.L.; Searle, J.B. Of mice and the 'Age of Discovery': The complex history of colonization of the Azorean archipelago by the house mouse (*Mus musculus*) as revealed by mitochondrial DNA variation. *J. Evol. Biol*. **2015**, *28*, 130–145.
31. Prager, E.M.; Sage, R.D.; Gyllensten, U.; Thomas, W.K.; Hübner, R.; Jones, C.S.; Noble, L.; Searle, J.B.; Wilson, A.C. Mitochondrial DNA sequence diversity and the colonization of Scandinavia by house mice from East Holstein. *Biol. J. Linn. Soc.* **1993**, *50*, 85–122.
32. Prager, E.M.; Tichy, H.; Sage, R.D. Mitochondrial DNA sequence variation in the eastern house mouse, *Mus musculus*: Comparison with other house mice and report of a 75-bp tandem repeat. *Genetics* **1996**, *143*, 427–446.
36. Hardouin, E.A.; Chapuis, J.L.; Stevens, M.I.; Van Vuuren, J.B.; Quillfeldt, P.; Scavetta, R.J.; Teschke, M.; Tautz, D. House mouse colonization patterns on the sub-Antarctic Kerguelen Archipelago suggest singular primary invasions and resilience against re-invasion. *BMC Evol. Biol.* **2010**, *10*, 325.
58. Bonhomme, F.; Orth, A.; Cucchi, T.; Rajabi-Maham, H.; Catalan, J.; Boursot, P.; Auffray, J.-C.; Britton-Davidian, J. Genetic differentiation of the house mouse around the Mediterranean basin: Matrilineal footprints of early and late colonization. *Proc. R. Soc. B* **2011**, *278*, 1034–1043.
60. Jones, E.P.; Jóhannesdóttir, F.; Gündüz, İ.; Richards, M.B.; Searle, J.B. The expansion of the house mouse into north-western Europe. *J. Zool.* **2011**, *283*, 257–268.
63. Jones, E.P.; van der Kooji, J.; Solheim, R.; Searle, J.B. Norwegian house mice (*Mus musculus musculus/domesticus*): Distributions, routes of colonization and patterns of hybridization. *Mol. Ecol.* **2010**, *19*, 5252–5264.
71. Linnenbrink, M.; Wang, J.; Hardouin, E.A.; Künzel, S.; Metzler, D.; Baines, J.F. The role of biogeography in shaping diversity of the intestinal microbiota in house mice. *Mol. Ecol.* **2013**, *22*, 1904–1916.
72. Gray, M.M.; Wegmann, D.; Haasl, R.J.; White, M.A.; Gabriel, S.I.; Searle, J.B.; Cuthbert, R.J.; Ryan, P.G.; Payseur, B.A. Demographic history of a recent invasion of house mice on the isolated Island of Gough. *Mol. Ecol.* **2014**, *23*, 1923–1939.
80. Gabriel, S.I.; Stevens, M.I.; Mathias, M.D.; Searle, J.B. Of mice and 'convicts': Origin of the Australian house mouse, *Mus musculus*. *PLoS ONE* **2011**, *6*, e28622.
98. Searle, J.B.; Jones, C.S.; Gündüz, İ.; Scascitelli, M.; Jones, E.P.; Herman, J.S.; Rambau, R.V.; Noble, L.R.; Berry, R.J.; Giménez, M.D.; Jóhannesdóttir, F. Of mice and (Viking?) men: phylogeography of British and Irish house mice. *Proc. R. Soc. B* **2009**, *276*, 201–207.
100. Belheouane, M.; Vallier, M.; Čepić, A.; Chung, C.J.; Ibrahim, S.; Baines, J.F. Assessing similarities and disparities in the skin microbiota between wild and laboratory populations of house mice. *ISME J.* **2020**, *14*, 2367–2380.
101. Gündüz, İ.; Auffray, J.-C.; Britton-Davidian, J.; Catalan, J.; Ganem, G.; Ramalhinho, M.G.; Mathias, M.L.; Searle, J.B. Molecular studies on the colonization of the Madeiran archipelago by house mice. *Mol. Ecol.* **2001**, *10*, 2023–2029.
102. Gündüz, İ.; Tez, C.; Malikov, V.; Vaziri, A.; Polyakov, A.V.; Searle, J.B. Mitochondrial DNA and chromosomal studies of wild mice (*Mus*) from Turkey and Iran. *Heredity* **2000**, *84*, 458–467.

103. Hauffe, H.C.; Panithanarak, T.; Dallas, J.F.; Piálek, J.; Gündüz, İ.; Searle, J.B. The tobacco mouse and its relatives: a "tail" of coat colors, chromosomes, hybridization and speciation. *Cytogenet. Genome Res*. **2004**, *105*, 395–405.
104. Ihle, S.; Ravaoarimanana, I.; Thomas, M.; Tautz, D. An analysis of signatures of selective sweeps in natural populations of the house mouse. *Mol. Biol. Evol.* **2006**, *23*, 790–797.
105. Jones, E.P.; Searle, J.B. Differing Y chromosome versus mitochondrial DNA ancestry, phylogeography, and introgression in the house mouse. *Biol. J. Linn. Soc.* **2015**, *115*, 348–361.
106. Nachman, M.W.; Boyer, S.N.; Searle, J.B.; Aquadro, C.F. Mitochondrial DNA variation and the evolution of Robertsonian chromosomal races of house mice, *Mus domesticus*. *Genetics* **1994**, *136*, 1105–1120.
107. Rajabi-Maham, H.A.; Orth, A.; Bonhomme, F. Phylogeography and postglacial expansion of *Mus musculus domesticus* inferred from mitochondrial DNA coalescent, from Iran to Europe. *Mol. Ecol.* **2008**, *17*, 627–641.
108. Renaud, S.; Ledevin, R.; Pisanu, B.; Chapuis, J.L.; Quillfeldt, P.; Hardouin, E.A. Divergent in shape and convergent in function: adaptive evolution of the mandible in Sub-Antarctic mice. *Evolution* **2018**, *72*, 878–892.
109. Sage, R.D.; Prager, E.M.; Tichy, H.; Wilson, A.C. Mitochondrial DNA variation in house mice, *Mus domesticus* (Rutty). *Biol. J. Linn. Soc.* **1990**, *41*, 105–123.
110. Storz, J.F.; Baze, M.; Waite, J.L.; Hoffmann, F.G.; Opazo, J.C.; Hayes, J.P. Complex signatures of selection and gene conversion in the duplicated globin genes of house mice. *Genetics* **2007**, *177*, 481-500.
111. Suzuki, H.; Nunome, M.; Kinoshita, G.; Aplin, K.P.; Vogel, P.; Kryukov, A.P.; Jin, M.L.; Han, S.H.; Maryanto, I.; Tsuchiya, K.; Ikeda, H. Evolutionary and dispersal history of Eurasian house mice *Mus musculus* clarified by more extensive geographic sampling of mitochondrial DNA. *Heredity* **2013**, *111*, 375–390.
